# Axon-bearing and axon-less horizontal cell subtypes are generated consecutively during chick retinal development from progenitors that are sensitive to follistatin

**DOI:** 10.1186/1471-213X-8-46

**Published:** 2008-04-25

**Authors:** Per-Henrik D Edqvist, Madelen Lek, Henrik Boije, Sarah M Lindbäck, Finn Hallböök

**Affiliations:** 1 Department of Neuroscience, Unit of Developmental Neuroscience, Biomedical Centre, Uppsala University, S-751 23, Uppsala, Sweden

## Abstract

**Background:**

Horizontal cells are retinal interneurons that modulate the output from photoreceptors. A rich literature on the morphological classification and functional properties of HCs in different animals exists, however, the understanding of the events underlying their development is still limited. In most vertebrates including chicken, two main horizontal cell (HC) subtypes are identified based on the presence or absence of an axon.

**Results:**

In this work we have molecularly characterized three HC subtypes based on Lim1, Isl1, GABA and TrkA, a classification that is consistent with three chick HC subtypes previously defined by morphology. The axon-bearing and axon-less HC subpopulations molecularly defined by Lim1 and Isl1, are born consecutively on embryonic day (E) 3–4 and E4–5, respectively, and exhibit temporally distinguishable periods of migration. Their relative numbers are not adjusted by apoptosis. A sharp decrease of high endogenous levels of the activin-inhibitor follistatin at E3 coincides with the appearance of the Lim1 positive cells. Extending the follistatin exposure of the HC retinal progenitor cells by injection of follistatin at E3 increased the number of both Lim1- and Isl1 positive HCs when analysed at E9.

**Conclusion:**

The results imply that the axon-bearing and axon-less HC subgroups are defined early and are generated consecutively from a retinal progenitor cell population that is sensitive to the inhibitory action of follistatin. The results are consistent with a model wherein added follistatin causes HC-generating progenitors to proliferate beyond the normal period of HC generation, thus producing extra HCs of both types that migrate to the HC layer.

## Background

During the development of the vertebrate retina, five types of neurons are born in a preserved sequential but overlapping order [1-3]. These are retinal ganglion cells, horizontal cells (HCs), amacrine cells, bipolar cells and photoreceptors. In the mature retina, these cell types are positioned within distinct laminas and can be further divided into subtypes based on morphology, neurochemical properties or function. At the end of the 19^th ^century, Ramón y Cajal recognized this complex lamination and morphological richness of the retina as well as identified retinal subtypes based on morphology. Today, neuronal subtypes are defined and distinguished by molecular and functional criteria rather than by morphology. Approximately 50–70 neuronal subtypes can be identified in the mature retina depending on the species [4,5].

Horizontal cells, which are the focus of this study, have been found in all vertebrate retinas from fish to man [6,7]. They are interneurons situated between photoreceptors and bipolar cells and form synapses with both cell types. In the chick retina, HCs form a distinct layer of cells that we will refer to as the horizontal cell layer (HCL), in the outermost part of the inner nuclear layer (INL). Two kinds of HCs are found in vertebrate retinas; axon-bearing and axon-less HCs [6,7]. An exception are retinas from several rodents, including mice, where only one HC type is identified [8]. In several species of fish, turtles, birds and mammals (including primates) a third HC subtype have been identified or suggested [4,6,9-12]. Regardless of the actual number of HC subtypes, the axon-bearing HC is present in all species and additional subtypes are axon-less [6]. The chick retina has three HC subtypes that have been identified based on their distinct morphological appearances [13,14]. One is the axon-bearing "brush-shaped" HC (also denoted H1 or Type I) and the two others are axon-less HCs, denoted "stellate" (or H2/Type II) and "candelabrum-shaped" (or H3/Type III) HCs. The brush-shaped HC axon-terminus mainly connects to rod photoreceptors, whereas the dendritic trees of all three HCs mainly form connections with cone photoreceptors [13,15,16]. This functional organisation appears to be conserved within vertebrate retinas [6,7].

A rich literature on the morphological and functional properties of HC subtypes exists but less is known about the developmental events underlying their generation. Recent studies have demonstrated that transcription factors such as Foxn4, Ptf1a and Prox1 are pivotal for the generation of the axon-bearing HCs in mice [17-21], and retinas deficient of the cell cycle inhibitors Ink4d and Kip1 overproduce those HCs [22]. In addition to intrinsic regulators like these, extrinsic factors are known to modulate different aspects of retinal development including proliferation, determination, differentiation and cell survival/death. Members of the transforming growth factor β/bone morphogenetic protein (TGFβ/BMP) family of growth factors are known to function as modulators during neuronal development [23] and an involvement of these molecules during the generation of retinal neurons have been suggested [24-30]. Follistatin which is an endogenous inhibitor of activin, TGF-β 1, GDF-11, and several other TGFβ/BMP molecules [27,31], is known to be expressed in the developing retina and pigment epithelium [24,25]. Interestingly, a recent study indicated that the generation of HCs may be affected by follistatin [28] since Prox1+ cells were found scattered at ectopic locations following virus mediated over-expression of follistatin in the developing retina.

The aim of this study was to gain further understanding of how and when HC subtypes are generated. We have molecularly characterized HC subtypes and found that the transcription factors Lim1 and Isl1 are expressed in axon-bearing and axon-less HC subtypes, respectively. We demonstrate that the axon-bearing and axon-less HC subpopulations are molecularly defined early in development, have different but overlapping birth dates, migrate to the HCL at different times and that their relative numbers are not adjusted by apoptosis. Endogenous follistatin expression is high during the earliest phase of retinal development, with levels dropping sharply at the time of HC generation. Moreover, we show that follistatin overexposure at the time of HC-generation causes an increase in both the early-generated Lim1+ axon-bearing and late Isl1+ axon-less HCs when analyzed at embryonic day 9/Hamburger & Hamilton stage 35 (E9, st35, [32]). Combined, these results imply that the two HC subgroups are defined early and are generated consecutively from a progenitor cell population that is sensitive to follistatin inhibition.

## Results

### Newly generated HCs express either Lim1 or Isl1 and constitute equally large populations

**Figure 1:**
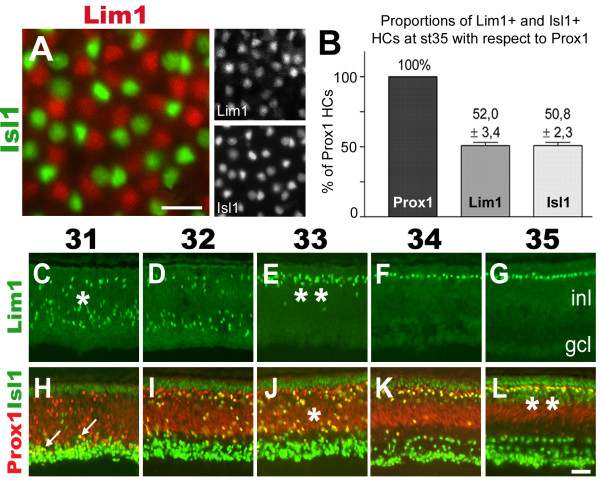
**Horizontal cells express either Lim1 or Isl1 in equally large proportions**. (A): The HCL of a flat-mounted normal retina section labelled for Lim1 (red) and Isl1 (green) reveals that the expression patterns do not overlap. (B): Quantification of HCs expressing Lim1 or Isl1 with respect to Prox1 at st35 (expressed as % ± s.d., n animals = 3). (C-L): Comparison of the labelling patterns of Lim1 (C-G, green) and Prox1 combined with Isl1 (H-L, red/green) in st31-35 (E7–E9) retinas reveal that Lim1 and Isl1 HCs migrate (*) and reach the HCL (**) at different time points. White arrows in H indicate double-labelled cells. Scale bars are 20 μm (A) and 40 μm (L, valid for C-L).

To identify immature HC subtypes several available antibodies against molecules known to be involved in neuronal determination were surveyed for reactivity to HCs. In addition to the transcription factors Prox1 and Lim1 that are expressed by immature HCs, Isl1 was identified as a novel transcription factor expressed in immature HCs. The result of the antibody survey is reported elsewhere [33]. First we analysed whether all HCs expressed Prox1 and Pax6, two well established HC markers, using flat-mounted retina sectioned through the HCL. The analysis showed that all cells located within the HCL expressed Prox1 and Pax6 (not shown). Stage 35 (E9) was selected for further analysis since the HCL is established within the INL at this age [34]. Retinas were analysed with respect to Prox1, Lim1 and Isl1 expression. The results showed that Lim1 and Isl1 immunoreactivities did not overlap, and that Lim1 and Isl1 immunoreactive HCs were mixed within the HCL (Fig. 1A). By counting double-labelled cells in the HCL of the central-most retina, the ratio of Lim1+ or Isl1+ HCs were determined to be 50/50 with respect to Prox1 (Fig. 1B). These results reveal that HCs are split into two equally large sub-groups based on the expression of Lim1 and Isl1.

### Isl1 HCs migrate one day after the Lim1 HCs

We have previously shown that HCs expressing Lim1 and Prox1 perform a bi-directional migration within the retina [34]. We investigated whether Isl1+ HCs performed a similar bi-directional migration by comparing the patterns of Lim1 (Fig. 1C–G), Prox1 (Fig. 1H–L, red) and Isl1 (Fig. 1H–L, green). The spatio-temporal pattern of Prox1+, Isl1+ double-labelled cells was similar, but delayed to, the Prox1+, Lim1+ double-labelled cells that have been shown to migrate bi-directionally (compare Fig. 1C with 1J and 1E with 1L, single and double asterisks). The first Prox1+, Isl1+ double-labelled cells were detected close to the prospective ganglion cell layer (GCL) at st31 (Fig. 1H, white arrows pointing at yellow cells). During subsequent stages numerous Prox1, Isl1 double-positive cells were seen scattered at various levels of the INL between st32 and st35 (Fig. 1H–L, yellow cells). Our analysis suggests that Prox1+, Isl1+ double-labelled cells initiate retrograde migration and reach the HCL one day after Lim1+ HCs.

### Isl1 HCs are born one day after Lim1 HCs

To investigate if the segregated migration patterns reflected a temporal difference in birth-dates or just a delay in migration between the two subgroups, we birth-dated the two HC populations using [^3^H]-dT. Stage 19–33 embryos received a single dose of [^3^H]-dT after which retinas were collected at st35 (E9) and processed for Lim1 and Prox1 immunocytochemistry and autoradiography. Stage 35 was analyzed since the mature 50/50 ratio of Lim1+ and Isl1+ HCs is set at this stage (see below and Fig. 1B). The fractions of Lim1+ or Prox1+ HCs also positive for [^3^H]-dT incorporation (silver grains) were determined for each injected stage (see Additional files 1 and 3). Since other retinal cell types in addition to HCs label for Isl1 [33], we inferred the birth-date of Isl1+ HCs by analysing the HCs expressing Prox1 but not Lim1.

**Figure 2:**
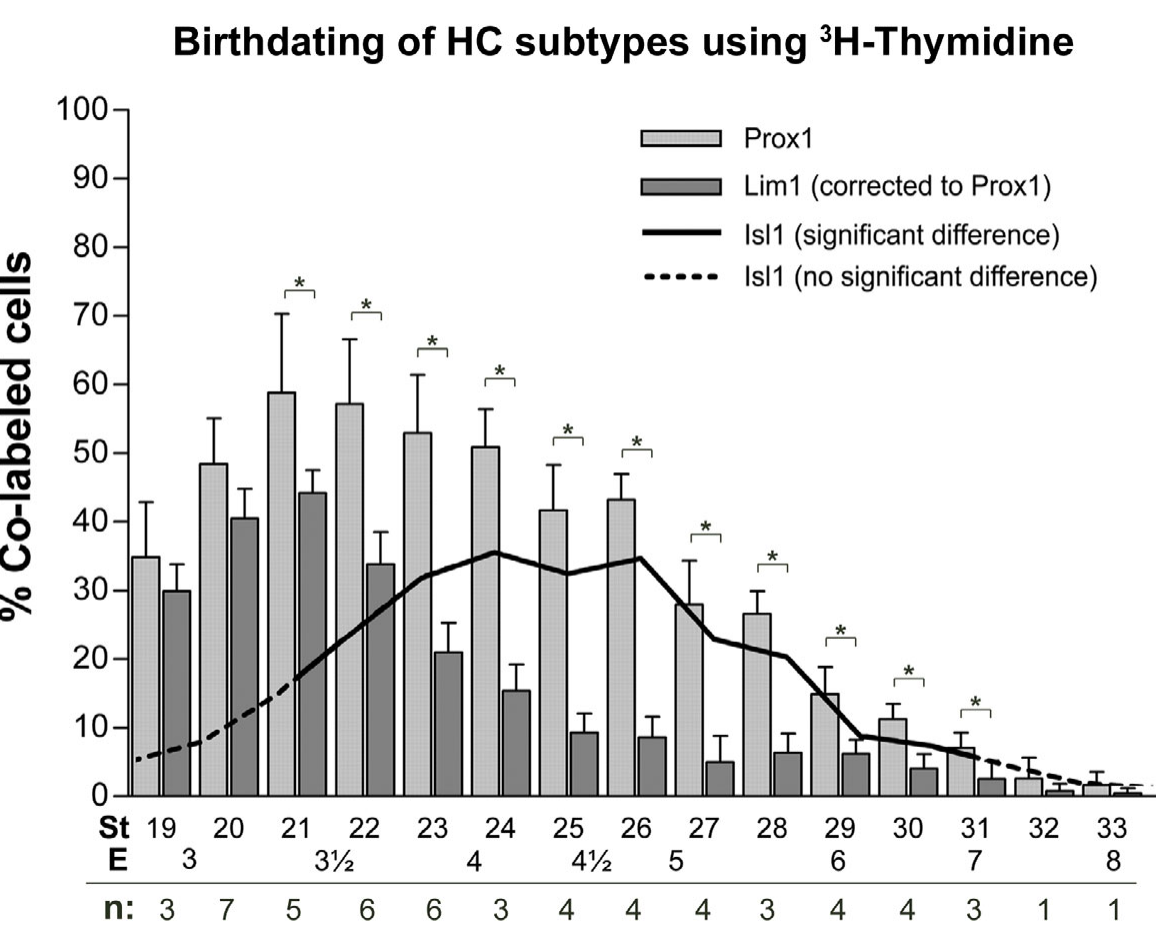
**Lim1 and Isl1 horizontal cell subgroups have different birth-dates**. Fractions of HCs positive for [^3^H]-dT incorporation combined with either Prox1 (light grey bars) or Lim1 (dark grey bars) analysed at st35 (expressed as % ± s.d., * p < 0.05 using the Student's t-test, n = number of animals that were analysed for each stage). The line indicates the difference between the Prox1 and Lim1 [^3^H]-dT-labelled fractions, and represents the birth curve for Isl1+ HCs. Solid portion of the line corresponds to significant differences. St: stage (of [^3^H]-dT injection), E: embryonic day.

Our data demonstrated that the HC population in the central retina (Prox1+ HCs, Fig. 2 light grey bars) was born between E3 and E6, in accordance with previous observations [1]. Furthermore, the birth of Lim1+ HCs peaked between E3 to E4 (Fig. 2, dark grey bars), whereas the birth of Prox1+, Lim1-negative HCs (deduced to be the same as the Isl1+ HCs) peaked between E4 to E5 (Fig 2, solid black line).

### Lim1 is expressed by axon-bearing and Isl1 by axon-less HCs

We investigated how the Lim1 and Isl1 expressing HC subgroups corresponded to the three HC subtypes previously identified by morphology [13]. Post-hatch day 7 (P7) retinas were analysed using confocal microscopy with antibodies against the known HC markers calretinin, GABA and TrkA [35-37] in relation to Lim1 and Isl1 expression. P7 retina was chosen since the morphology of all three HC subtypes were difficult to assess at earlier stages using immunohistochemistry.

**Figure 3:**
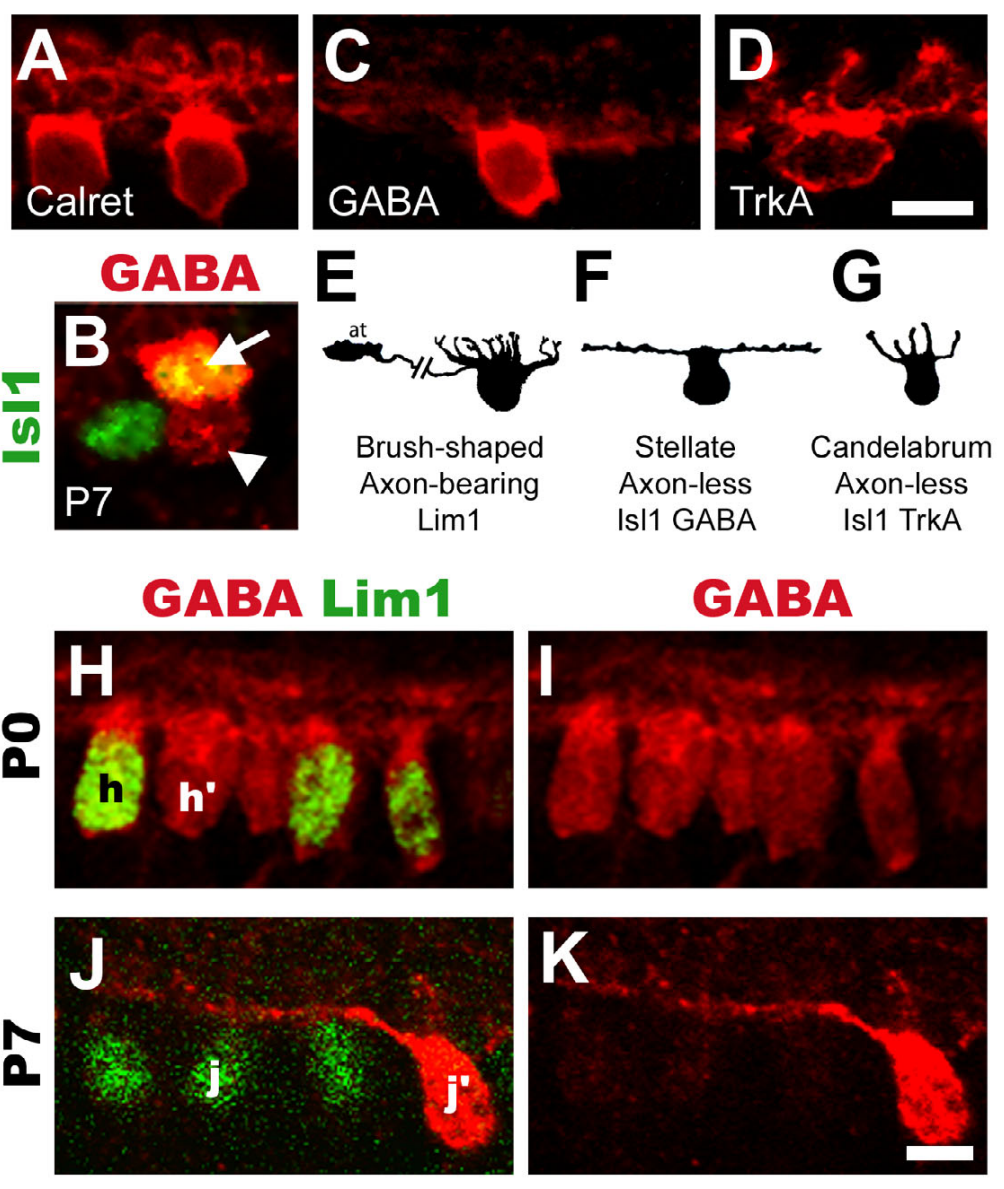
**Confocal analysis of horizontal cell morphology and changes in GABA expression levels after hatch**. (A): Axon-bearing, brush-shaped HCs visualised by calretinin. (B): Flat-mounted P7 retina. GABA antibodies (red) label a fraction of Isl1+ HCs intensely (green, arrow, co-expression is yellow), and label Lim1+ HCs to a lesser extent (arrowhead). (C): Axon-less stellate HCs visualized by intense GABA labelling. (D): Axon-less candelabrum-shaped HCs visualised by TrkA. (E-G): Morphology of chick HCs as they appear in Gallego, 1986 [6] with names and molecular attributes denoted below. (H-K): P0 (H-I) and P7 (J-K) HCs labelled for GABA (H-K, red) and Lim1 (H and J, green). Uniform GABA levels are found in both Lim1+ and Lim1-HCs at P0 (compare h with h') whereas Lim1+ HCs express low levels of GABA at P7 (compare j with j'). Scale bars are 5 μm (valid for A-D and H-K).

**Figure 4:**
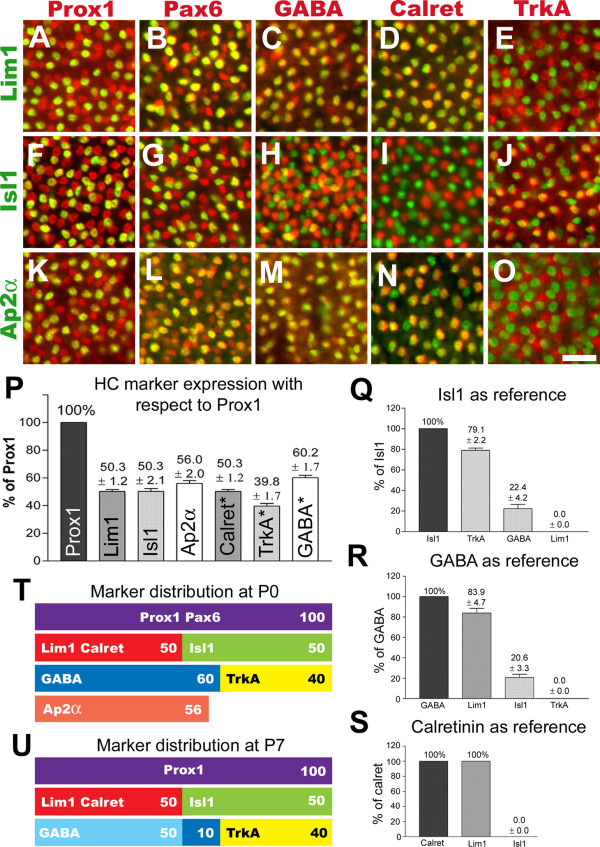
**Horizontal cell subtypes express different sets of markers**. (A-O): The horizontal cell layer of flat-mounted P0 retinas labelled with Lim1 (A-E, green), Isl1 (F-J, green) and Ap2α (K-O, green), combined with Prox1 (A, F, K, red), Pax6 (B, G, L, red), GABA (C, H, M, red), calretinin (D, I, N, red) and TrkA (E, J, O, red). Double positive cells appear yellow. (P-S): Calculated fractions (expressed as % ± s.d., n animals = 3) of HCs co-expressing various markers with respect to Prox1 (P), Isl1 (Q), GABA (R) and calretinin (S). Asterisks in P denote that these fractions were calculated using known fractions of other markers. (T-U): Distribution of HC markers at P0 and P7. Scale bar is 20 μm (valid for A-O).

Calretinin and Lim1 were co-expressed in HCs that had morphology similar to the brush-shaped, axon-bearing HC (compare Fig. 3A with 3E, Fig. 4D). Weak GABA labelling was detected in Lim1+ HCs (Fig. 3B, arrowhead) while intense labelling was seen in a small subset of Isl1+ HCs (Fig. 3B, arrow). This intense labelling was found in HCs with morphology similar to the axon-less stellate HC (compare Fig. 3C with 3F). TrkA was found exclusively in the majority of Isl1+ HCs (see below) that had morphology similar to axon-less candelabrum-shaped HCs (compare Fig. 3D with 3G).

### GABA decreases in axon-bearing HCs from hatch to P7

GABA is considered to be the main neurotransmitter in these inhibitory interneurons. Our results as well as others show that GABA is present in a subset of HCs [38,39]. We compared GABA labelling in HCs from st44 (E18), P0, P4 and P7 chicks. At st44 (not shown) and P0, GABA labelling was uniform in both Lim1+ and Isl1+ HCs (compare Fig. 3H–I, h and h') while at P4, a difference was notable (not shown). At P7 GABA antibodies just weakly labelled Lim1+ HCs whereas a subset of Lim1-negative HCs was strongly labelled by GABA (Fig. 3J–K, j and j'). In summary, GABA levels gradually change in two of the subtypes after hatching with high levels remaining in stellate Isl1+ HCs, whereas GABA-levels in axon-bearing HCs decrease.

### Horizontal cell subtype population sizes

Horizontal cells from newly hatched P0 chick retinas were labelled using combinations of antibodies directed against Lim1, Isl1, Ap2α, Prox1, Pax6, GABA, calretinin and TrkA. All markers except Isl1 and Ap2α are previously well established HC markers [18,34,36,37,40,41]. In the P0 chick retina, Prox1 and Pax6 antibodies labelled all HCs. Consistent with their expression at st35, Lim1 and Isl1 did not overlap (not shown). Lim1 and Isl1 were found in fractions of Prox1, Pax6 and GABA labelled HCs (Fig. 4A–C, F–H). Calretinin was exclusively expressed in Lim1+ HCs (compare Fig. 4D with 4I) and TrkA was expressed in a fraction of Isl1+ HCs (compare Fig. 4E with 4J). GABA and TrkA labelling did not overlap (not shown). Ap2α was expressed in all axon-bearing and some axon-less HCs (Fig. 4K–O). At P0, Ap2α was expressed in the majority of GABAergic HCs (Fig. 4M), in all calretinin+ HCs (Fig. 4N), but not in TrkA+ HCs (Fig. 4O).

We quantified the fractions of cells single- or double-positive for these markers in the central retina (in direct proximity to the optic nerve exit), and found that at P0, 50% of all HCs expressed either Lim1 or Isl1, Ap2α was expressed in 56% of all HCs, and GABA and TrkA was expressed by 60% and 40% of all HCs, respectively (Fig. 4P–T). About 20% of the axon-less (Isl1+) HCs were labelled for GABA, and the remaining Isl1+ cells for TrkA (Fig. 4Q). Of all HCs expressing GABA at P0, 80% were axon-bearing (Lim1+) HCs, whereas the remaining 20% were of the axon-less stellate HC type (Fig. 4R). Quantification with respect to calretinin confirmed the previous observations of calretinin expression in axon-bearing HCs (Fig. 3A and 4S).

Our data reveal the existence of two complementary divisions of the HC population where Lim1 and Isl1 split the HC population 50/50, and GABA and TrkA split the population 60/40. Combined, these divide the HC population into three distinct groups where axon-bearing Lim1+ HCs constitute 50%, axon-less stellate HCs expressing Isl1 and GABA constitute 10% and axon-less candelabrum HCs expressing Isl1 and TrkA constitute 40% of the HC population (Figs 4T–U, see also 3E–G).

### Apoptosis does not affect the Prox1+ HC population

**Figure 5:**
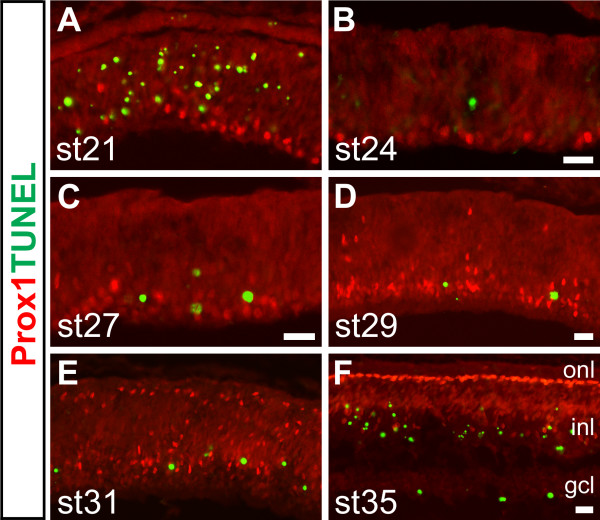
**Horizontal cells are not affected by apoptosis**. (A-F): Stage 21 (A), 24 (B), 27 (C), 29 (D), 31 (E) and 35 (F) retinas stained for TUNEL (green) and Prox1 (red) reveal no overlap between the two signals. Scale bars: 20 μm (B and F are valid for A-B and E-F).

To investigate whether apoptosis sculpted the relative proportions of HC subtypes during early retinal development, we performed TUNEL staining combined with Prox1 antibody labelling in all developmental stages ranging from st20 to st36 as well as in selected later embryonic and post-hatch stages. We were unable to detect TUNEL stained Prox1+ cells in any of the early stages we investigated (Fig. 5A–F). At later retinal stages, a double-stained cell could occasionally be found in the extreme peripheral retina, but not in the early developing retina nor in the central retina. TUNEL and Isl1+ double-labelled cells belonging to other neuronal populations were observed (not shown).

### The onset of Lim1 expression is preceded by a drastic wave-like decrease of endogenous follistatin expression

**Figure 6:**
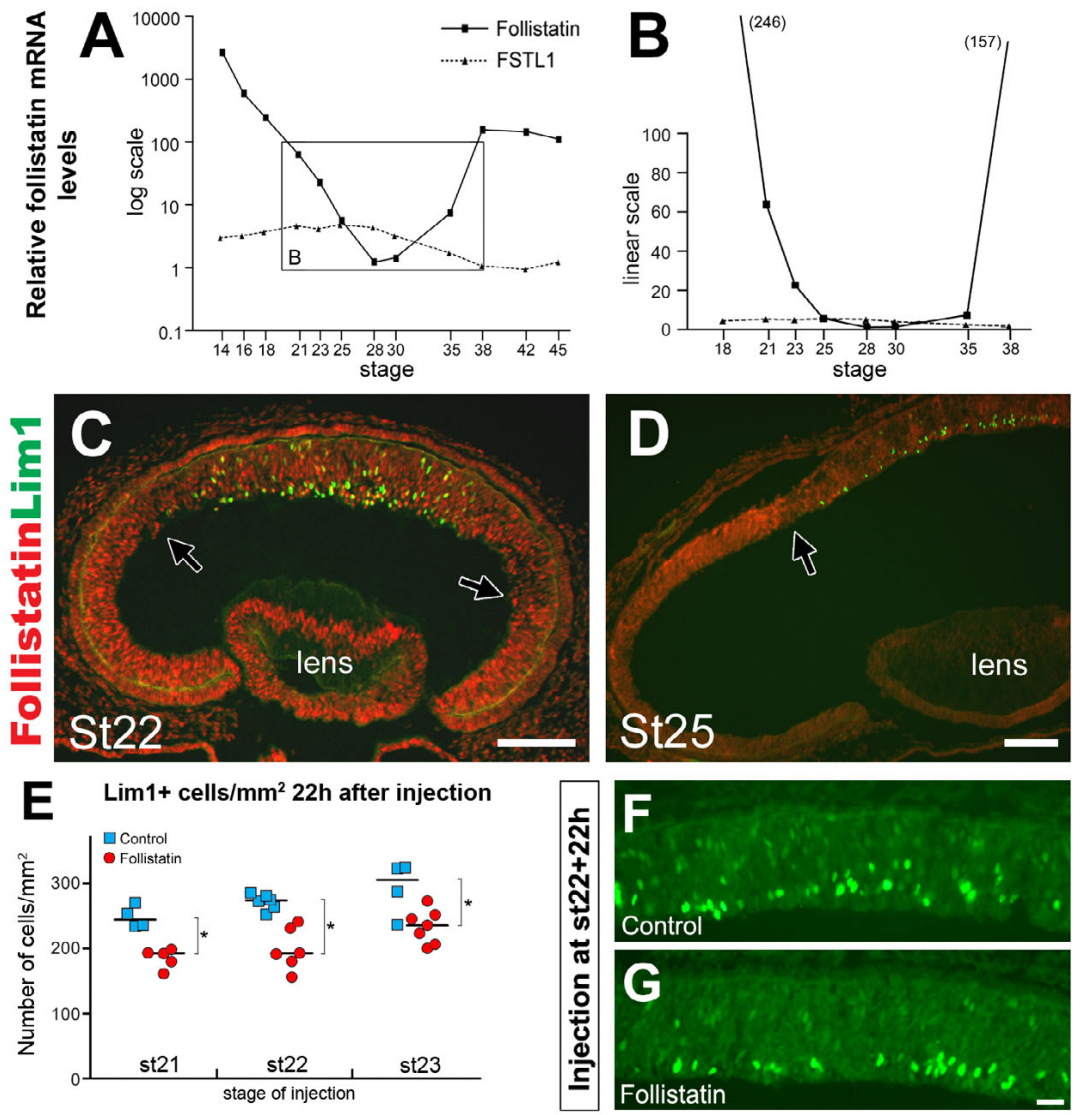
**Decreased follistatin expression coincides with the generation of horizontal cells**. (A-B): Follistatin (solid line) and follistatin-like 1 (FSTL1, dashed line) mRNA levels during retinal development relative the lowest value respectively which is set to 1 (follistatin: 1 = st28; FSTL1: 1 = st42). The X-axis denotes the embryonic stages analysed and the Y-axis denotes the relative mRNA levels in log scale (A) or linear scale (B). Numbers in parenthesis in B denote the relative follistatin mRNA level-value at st18 and 38, respectively. (C-D): Follistatin immunoreactivity (red) in the developing retina at st22 (C) and st25 (D) reveal a central-to-peripheral gradient of follistatin expression. Highest levels are expressed peripherally and sharp boundaries between high and low expression are evident (black arrows). The onset of Lim1 expression (green) is trailing the wave-front of decreasing follistatin expression. (E): 22 h after follistatin treatment at st21, st22 or st23, the number of Lim1+ cells decreases by approximately 20–25%. Lim1+ cells were counted in 20× visual fields and expressed as Lim1+ cells/mm^2 ^retina. Blue squares indicate the means of individual control treated animals and red circles indicate the means of individual follistatin-treated animals. The medians are indicated by a black line, * denote significant differences (p < 0.05, Mann-Whitney test). F-G: Lim1 expression (green) in central retina 22 h after control (F) or follistatin (G) treatment in st22 embryos. Scale bars are 100 μm (C-D), and 20 μm in G (valid for F-G).

Previous results have shown that over-expression of follistatin in the developing retina results in ectopic cells expressing Prox1 [28]. This observation prompted us to further investigate the HC generation with respect to follistatin. We analyzed the follistatin mRNA expression in total retina from st14 to st45 embryos using qRT-PCR. The levels were normalized to the house-keeping genes β-actin and TATA-box binding protein. By far, the highest levels of follistatin mRNA were in the optic cup and early eye (Fig. 6A, note logarithmic graph scale, see also Additional file 3). The mRNA levels dropped sharply until reaching relatively very low levels between st23 and st30 (Fig. 6B, note linear graph scale). The levels increased again after st35. For comparison we analyzed the related factor follistatin-like 1, which in contrast to follistatin was not developmentally regulated (Fig. 6A–B). The drop in follistatin mRNA levels between stages 18 and 25 (Fig. 6C and 6D) coincides with the birth of Lim1+ HCs (Fig. 2).

Lim1 expression commences in the central retina at st19-20 [33] and extends peripherally as development progresses. At the early developmental stages, follistatin immunoreactivity was strong in retina, lens and pigment epithelium. With increasing age, a central to peripheral gradient of different strengths of follistatin immunoreactivity in the retina became progressively more obvious, with the strongest immunoreactivity levels located in the periphery (Fig. 6C and 6D, black arrows). The onset of Lim1 expression followed close behind the decrease of follistatin immunoreactivity as it moved towards the periphery (Fig. 6C and 6D). As development progressed (E4–E8), the endogenous expression of follistatin in the central retina became restricted to cells located in the putative GCL, whereas the expression in the central-outer retina gradually waned (not shown). By st34 (E8), follistatin immunoreactivity was restricted to the GCL and to cells located on the inner rim of the INL (see Additional file 2).

### Injection of follistatin at E3 decreases the number of newly generated Lim1+ cells

As retinal development progressed in a central to peripheral fashion, we observed that decreasing levels of follistatin immunoreactivity preceded the onset and expansion of Lim1 expression. This inverse correlation between HC generation and high follistatin levels suggested that follistatin overexposure may interfere with the generation of Lim1+ cells. We injected st21, st22 and st23 embryos with follistatin or vehicle and analysed the number of Lim1+ cells in the central retina 22 h after the injection. The follistatin bolus injection caused a significant 20–25% reduction in the number of Lim1+ cells per mm^2 ^retina in all stages tested (p < 0.05, Mann-Whitney test, Fig. 6E and 6F–G, see also Additional file 3).

**Figure 7:**
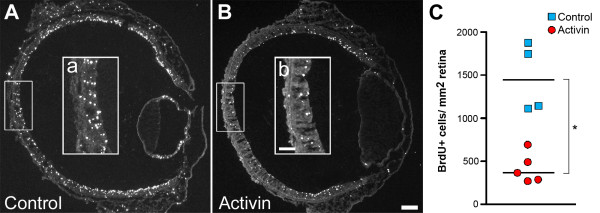
**Decreased BrdU incorporation following activin treatment**. BrdU incorporation was assayed in st23 activin treated retinas (A) and control injected retinas (B) using immunohistochemistry. After activin treatment, BrdU incorporation was markedly reduced compared to controls (compare a and b). (C): When quantified, this reduction was found to be significant (* p < 0.05, Mann-Whitney test). Blue squares indicate the means of individual control treated animals and red circles indicate the means of individual activin-treated animals. The medians are indicated by a black line. Scale bars B and b are 100 μm and 50 μm, respectively (valid for A-B and a-b).

The main target for the inhibitory action of follistatin is activin [31,42], which is expressed in the developing retina [24] and is known to decrease cell proliferation in many cell types and tissues [42]. We therefore hypothesised that the reduction of newly generated Lim1+ cells by the added follistatin, was an effect on the Lim1-generating progenitor cells. The inhibitory action of follistatin would then keep the progenitor cells proliferating and thus delaying cells from exiting the cell cycle and turning on Lim1 expression. This would lead to fewer newly generated Lim1+ cells compared to controls. To test this we added BrdU 5 h after either follistatin or activin injections to mark cells passing through S-phase. The number of BrdU cells was analyzed after a total of 22 h. The activin injections caused a robust decrease in BrdU incorporation compared to controls (Fig. 7A–C, see also Additional file 3) while the follistatin injections did not lead to any conclusive changes (data not shown). This indicates that activin treatment caused either an overall decreased proliferation rate or caused retinal progenitor cells (RPCs) to undergo premature cell cycle withdrawal. This finding indirectly support the hypothesis that the bolus dose of follistatin delays the onset of Lim1 expression by keeping progenitor cells in a proliferative state.

### Injection of follistatin at E3 affects retinal histology at E9

**Figure 8:**
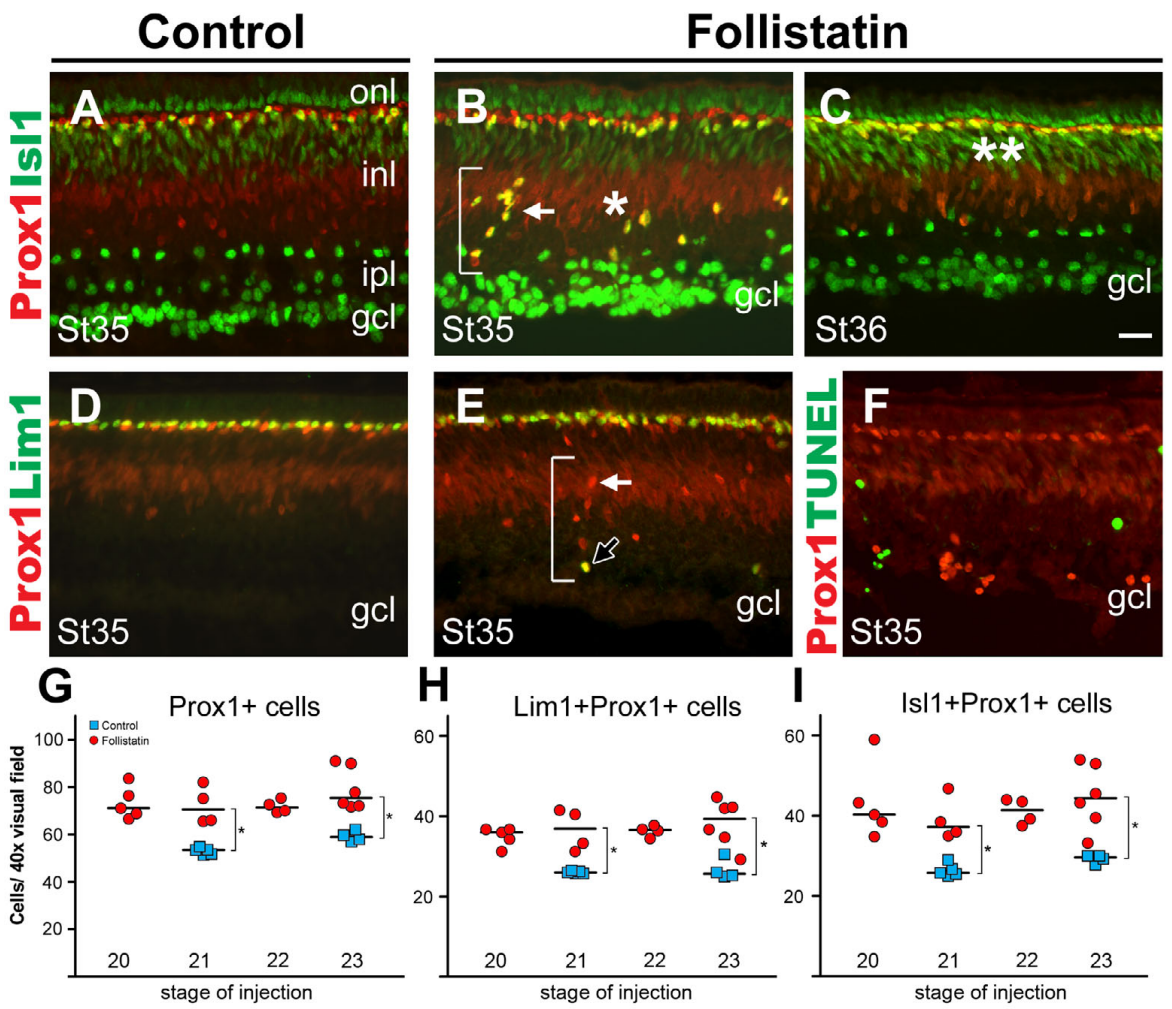
**Follistatin treatment at E3 increase the number of horizontal cells at E9**. (A-F): Retinal cross-sections stained for Prox1 (A-F, red) combined with Isl1 (A-C, green), Lim1 (D-E, green) or TUNEL (F, green). Co-labelled cells appear yellow. Compared to st35 control retinas (A and D) follistatin treated retinas (B, C and E) have ectopic Prox1+ cells in the inner retina which are often arranged in columns (B and E, brackets). These ectopic Prox1+ cells are primarily of the Isl1+ HC subtype (white arrows), although some are Lim1+ (E, black arrow). (C): At st36, ectopic HCs have migrated to the HCL (compare double asterisks with single asterisk in B). (F): St35 Prox1 and TUNEL-staining reveal no overlap. (G-I): Quantification at st35 of the number of HCs in 40× visual fields following follistatin treatment at various E3-stages. Blue squares indicate the means of individual control treated animals and red circles indicate the means of individual follistatin-treated animals. The medians are indicated by a black line. Follistatin causes a significant increase in the number of HCs (Prox1+) and HC subtypes (Lim+ or Isl1+ HCs) at all stages, compared to controls (* p < 0.05, Mann-Whitney test, see also Additional file 3). Scale bar is 20 μm (valid for A-F).

Effects on HCs can definitely be monitored first at st35 (E9), the time when both Lim1+ and Isl1+ HCs have migrated to the HCL. We performed a series of injections of follistatin into eyes of embryos ranging from st18-31. Analyses of Prox1, Lim1 and Isl1 immunoreactivity were then carried out at st35 (E9). We found that the structure of the inner plexiform layer was frequently distorted in embryos injected at early stages up to st24, but that no obvious phenotype could be observed from st26 and beyond (not shown). The typical follistatin-histology in our experiment at st35 is consistent with Moreira and Adler's observations [28], and was characterized by a thinner inner plexiform layer (Fig. 8, compare A with B and C) and ectopic Prox1+ cells either scattered or arranged in columns spanning the GCL and/or INL (Fig. 8B and 8E, brackets). This phenotype served as our criterion to determine if a retinal region had been affected by follistatin, and images from such regions were acquired and used for further analysis.

### Follistatin produces ectopic horizontal cells that migrate back to the HCL

The expression of Prox1, although at ectopic locations in the st35 (E9) retina, hinted that the cells were HCs. We wanted to know whether the ectopic Prox1+ cells were in fact HCs and investigated if they in addition to Prox1 expressed Lim1 or Isl1. Embryos were analysed with respect to HC development at st35. Some of the retinas were analysed one day later at st36 (E10). This relatively long post-injection time of 6–7 days was chosen in order to ensure the correct identification and quantification of HC subtypes, based both on their marker expression and localization in the retina.

Double immunohistochemistry for Prox1, Lim1 and Isl1 (Fig. 8A–E) showed that the ectopic Prox1 cells seen at st35 expressed either Lim1 or Isl1, indicating that ectopic Prox1+ cells were bona fide HCs. Consistent with our previous results, we did not observe Lim1 and Isl1 co-expression in control or follistatin-treated retinas (not shown). The ectopic cells in the st35 retina were predominantly of the Prox1, Isl1 double positive HC subtype (Fig. 8B and 8E, white arrows) and only a fraction of the ectopic Prox1+ cells expressed Lim1 (Fig. 8E, black arrow). One day later at st36/E10, ectopic HCs could not be found (Fig. 8C). Since the total number of cells double-positive for Prox1 and Lim1 or Prox1 and Isl1 (i.e. the ectopic cells and the ones present in the HCL) were similar in st35 and st36 retinas (data not shown), and apoptosis was not detected in normal or ectopic Prox1+ cells as shown by TUNEL (Fig. 8F), it was concluded that the st35 ectopic HCs had migrated back to the HCL at st36 (compare Fig. 8B and 8C, single and double asterisks) following a normal although delayed migration route.

### Injections of follistatin at E3 increase the number of HCs at E9

When analysing regions containing ectopic Prox1+ cells it appeared as if the number of Prox1+ HCs had increased. This was in contrast to the results obtained for Lim1+ cells 22 h after follistatin administration. We injected follistatin in a series of embryos at the phase of HC generation. As shown by the HC birth-dating experiments (Fig. 2), st20 and st23 correspond to the initial phases of the generation of the two HCs populations, respectively. This period also corresponds to when the endogenous follistatin levels drop (Fig. 6A–B). Controls were injected with vehicle at st21 or st23. We counted cells double-positive for Prox1 and Lim1, and Prox1 and Isl1 at st35, in retinas treated with follistatin at st20, 21, 22 or st23 and each stage was then compared individually with controls treated with vehicle at st21 or st23.

If the st20 treatment preferentially had effects on the early Lim1 population and not on the later Isl1 population, while the st23 treatment had effects on the late population, it could be hypothesized that the Lim1 and Isl1 HC subtypes are generated from separate progenitor cells. Conversely, if the early follistatin treatment also had effects on the late population it is likely that both subtypes are generated from a common pool of progenitor cells that is affected by the follistatin inhibition and expanded.

We found that follistatin treatment at all 4 stages caused a significant increase in the total number of HCs (Prox1+) as well as significant increases in both the number of Lim1+ and Isl1+ HC subtypes compared to controls (Fig. 8G–I, see also Additional file 3). With respect to the number of Lim1+ or Isl1+ cells in follistatin-treated animals, we found no difference between the early and late injection stages (Fig. 8G–I). Over all, the increase in the total number of Prox1+ cells was approximately 30%, for Lim1, Prox1 double positive cells approximately 40%, and for Isl1, Prox1 double positive cells approximately 50% (means of the 4 stages, see also Additional file 3). Thus, our data showed that follistatin overexposure at st20, 21, 22 or st23 caused an over-production of both the Lim1+ and the Isl1+ HC subtypes when analyzed at st35, regardless of the stage of treatment.

### Follistatin treatment and the effects on other retinal cell types

**Figure 9:**
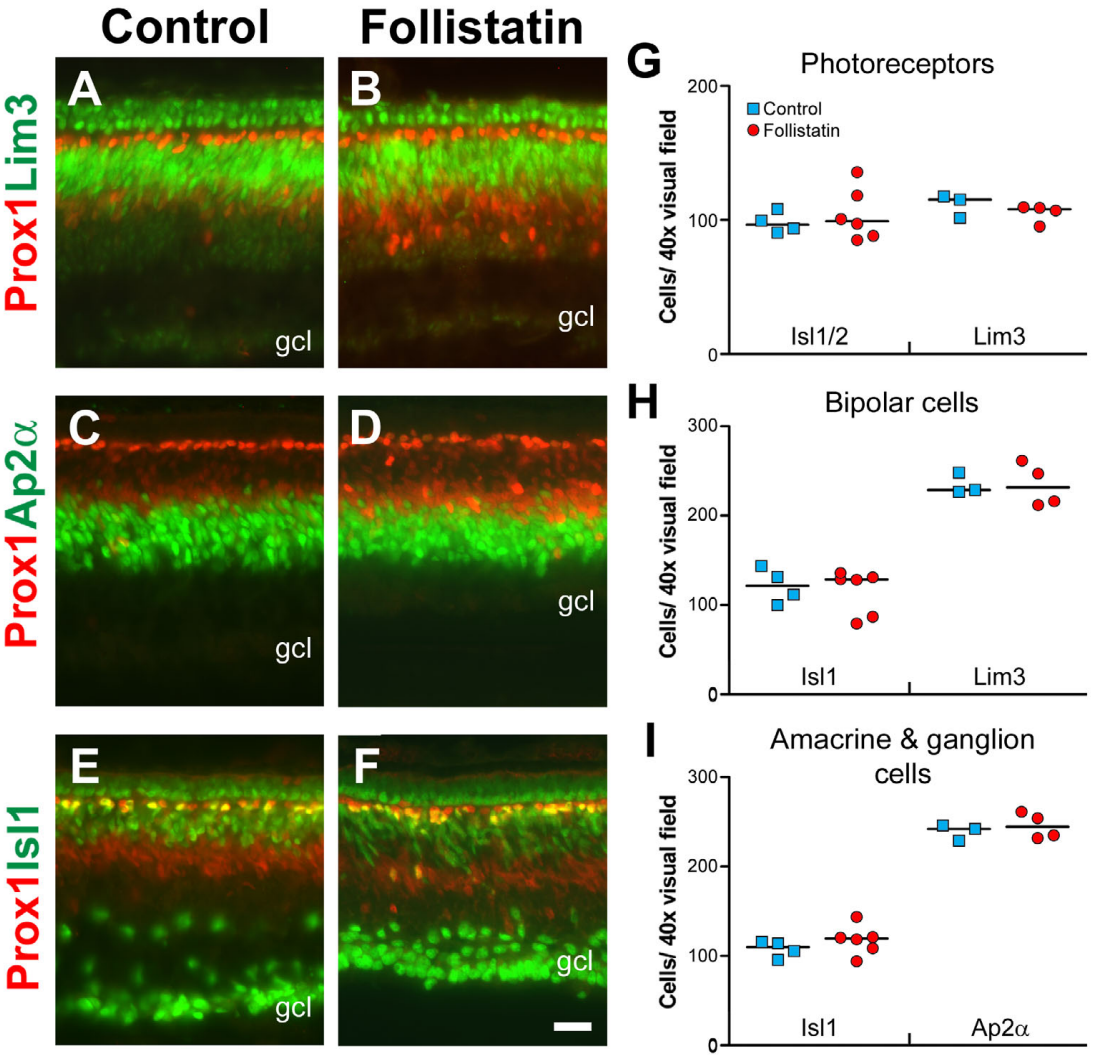
**Effects on other cell populations following follistatin treatment**. Control (A, C, and E) or follistatin-treated retinas (B, D, and F) stained for Prox1 (A-F, red) and Lim3 (A-B, green), Ap2α (C-D, green) and Isl1 (E-F, green). All injections were made at st23 and analysed at st35. With respect to the thickness of immuno-labelled bands, all markers appear grossly normal after follistatin injection. (G-I): Quantification of immuno-labelled cells in 40× visual fields. Blue squares indicate the means of individual control treated animals and red circles indicate the means of individual follistatin-treated animals. The medians are indicated by a black line. Using the above markers, no significant differences (p < 0.05, Mann-Whitney test) were found between control or follistatin treated retinas with respect to the number of photoreceptors, bipolar cells, amacrine cells or ganglion cells. Note that the Isl1+ cells quantified in 9I include both Isl1+ ganglion and amacrine cells. Scale bar is 20 μm (valid for A-F).

We analysed whether follistatin treatment at st23 influenced the generation of cell types other than HCs by quantifying the number of cells carrying markers against photoreceptors (Lim3, Isl1/2), amacrine cells (Ap2α), bipolar cells (Lim3, Isl1) and ganglion cells (Isl1) in st35 retinas [33]. We did not detect any differences in the number of immunoreactive cells in the outer nuclear layer after follistatin treatment using Lim3 (Fig. 9A–B) or Isl1/2 antibodies (Fig. 8A–C and 9E–F). The cells in the outer nuclear layer labelled by the Isl1/2 antibody most likely correspond to Isl2+ cells, since labelling with another Isl1 specific antibody did not produce immunoreactivity in these cells (not shown). In the INL, the bipolar cell population marked by Lim3 and Isl1 (Fig. 9A–B and 9E–F), and the amacrine cell population marked by Ap2α (Fig. 9C–D) were not affected by the follistatin treatment (Fig. 9H–I). In the GCL we did not detect any significant differences in the number of Isl1+ cells compared to controls (Fig. 8A–C and 9E–F, I). Noteworthy is that due to loss of inner plexiform layer thickness following follistatin treatment, we could not distinguish Isl1+ ganglion cells from Isl1+ amacrine cells normally present in the inner nuclear- and plexiform layers (compare Fig. 8A and 8B). Therefore, the Isl1 cell-counts in figure 9I include both these cell types. These data are also summarized in Additional file 3. These data suggest that under these experimental conditions, HCs is the only cell type that is affected by the follistatin treatment at st23.

## Discussion

### Horizontal cell subtypes and their maturation

In this work we have demonstrated that HCs are generated as two equally large and distinct subgroups during early retinogenesis. One subgroup, corresponding to the axon-bearing HC, expresses Lim1 whereas the other subgroup, corresponding to the axon-less HCs, expresses Isl1. The axon-less (Isl1+) HC subpopulation can be further subdivided in two HC subtypes. The Lim1 and Isl1 HC subgroups are generated and then migrate bi-directionally in the developing retina during successive periods. Moreover, we show that retinal progenitor cells that are able to generate both Lim1 and Isl1 HCs respond to follistatin overexposure at E3. The treatment results in significant increases in both Lim1 and Isl1 HC subtypes at E9. This effect is specific to HCs, and is best explained by a sustained proliferation of HC-generating progenitor cells.

We have demonstrated two types of axon-less HCs, the stellate and candelabrum-shaped HCs that in addition to Isl1, express GABA and TrkA, respectively. The axon-bearing brush-shaped HCs express calretinin in addition to Lim1. We quantified the proportions of the HC subtypes in the central P0 chick retina and found that the axon-bearing brush-shaped HC and the axon-less stellate and candelabrum-shaped HCs constituted 50%, 10% and 40% of the HC population, respectively. These findings are consistent with previous reports on chick HCs [6,13,38,39,43]. However, in slight contrast to our findings one recent report demonstrated that in P7–P21 chick retina a small fraction of Isl1+ HCs express calretinin [38], an observation that is not supported by our P0 retinal preparations and it is therefore possible that a subset of Isl1+ HCs turn on calretinin expression after hatching. The same study also identified what was classified as a fourth HC subtype based on their analysis of HC markers. A fourth HC subtype is also described in the pigeon retina based on morphological criteria [12]. Our results support the classical division of chick HCs into three main subtypes [6,13] but the actual number and extent of heterogeneity in HC subtypes in the chick retina is an unsettled issue.

As the retina undergoes late maturation, synapse rearrangement and changes in the expression levels of various proteins are known to occur [44-46]. We found that GABA was initially present uniformly in all GABA-containing HCs, but that GABA levels progressively decreased in axon-bearing HCs and increased in axon-less stellate HCs after hatch. This implies that GABA has a role in adult stellate HCs but not in adult axon-bearing cells. Rather, GABA may have a transient function in the developing axon-bearing HCs. This is in agreement with a role for GABA during synapse maturation when connections between HCs and photoreceptors or bipolar cells are established [47]. Other studies have reported changes in GABA levels during phenotypic maturation of HCs in rats, rabbits, guinea pigs and humans [48-51].

### Early development of HC subtypes

**Figure 10:**
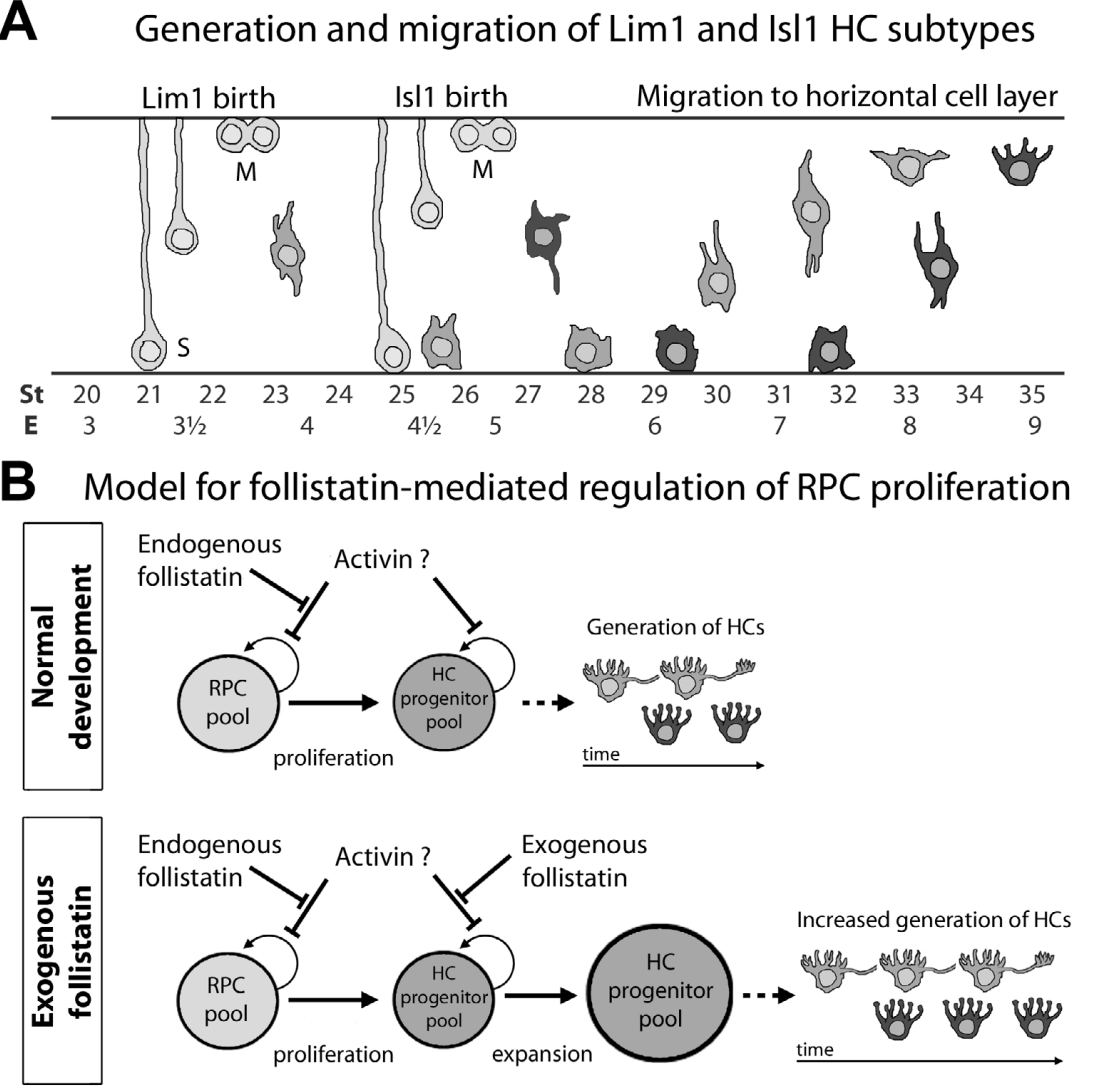
**Schematic summary of horizontal cell development**. (A): Schematic figure of the generation and migration of Lim1+ and Isl1+ HCs. S and M denote stages of the cell cycle. Position of M-phase cells is according to the classical view, however see also Godinho et al [56]. (B): Proposed model for the effects of follistatin on RPCs during retinogenesis. During normal development, high follistatin levels stimulate the proliferation of RPCs by preventing e.g. activin (or related factors) to decrease or inhibit proliferation. As follistatin decreases over time, RPCs specialize to generate e.g. HC progenitors. Under the influence of exogenous follistatin, again preventing activin (or related factors) from controlling cell cycle withdrawal, proliferation in the HC generating progenitor pool is stimulated and therefore, more HCs than normal are eventually generated.

Most classes of retinal neurons undergo cell death during development [52]. Photoreceptors and HCs are likely exceptions to this rule [53] and it has been shown that HCs do not undergo apoptosis during later stages of development [54,55]. We investigated apoptosis in HCs using TUNEL during early (Fig. 5) and late retinal development (not shown), but could not find evidence to suggest that the HC population size was adjusted by apoptosis. Moreover, Lim1+ HCs have been shown to migrate bi-directionally across the retina [34] and in this study we show that the Prox1, Isl1 double positive HCs followed a similar but delayed pattern, migrating approximately one day later (compare asterisks in Fig. 1C–L, Fig. 10A). Hence, we concluded that Isl1+ HCs also undergo bi-directional migration.

We investigated whether the two HC subpopulations marked by Lim1 or Isl1 were born simultaneously or consecutively by performing a birth-dating analysis using [^3^H]-dT incorporation. Our results revealed that although the two birth-curves overlapped, the majority of Lim1+ HCs were born between E3 and E4 whereas the majority of the HCs expressing Isl1+ were born between E4 and E5 (Fig. 2). However, since the earliest stage when Isl1+ HCs can be positively identified using Prox1 is st31 (Fig. 1H, arrows), the lack of earlier HC subtype markers makes it difficult to state with certainty that HC progenitors that give rise to the Isl1+ HCs are in fact born into an Isl1-fate (and not born as Lim1+ cells). However, the birth-curve of the Isl1+ HCs is concomitant with the appearance of Prox1+, Lim1-negative cells in the retina between E5.5 and E7 [34]. These Prox1-single positive cells likely correspond to the Isl1+ HC fraction before the actual onset of Isl1 expression. Thus, the birth-dating results inferring that Isl1+ HCs undergo their last S-phase around E4–5, which is just prior to the appearance of Prox1-single positive cells at E5.5, supports a scenario where the late HC subgroup identity is established at the time of cell cycle withdrawal. In addition, we do not find any Isl1 and Lim1 double labelling in HCs (Prox1+ cells) after st31. Godinho et al recently used live imaging in the zebrafish retina to show that the mitoses generating HCs occurred in the INL [56]. Combined, these data opens up the possibility that HCs in chick are generated as cells committed into distinct fates (Lim1/early or Isl1/late), and that the generation of these subgroups is temporally separated.

The results from a recent lineage study of chick HCs suggested that there was no significant difference in the birth order among three identified HC subtypes [57]. Our birth-dating experiment only considered HCs in the very central part of the retina and as the generation of HCs proceed further peripherally in the retina the temporal overlap of the generation may be larger. This may explain the discrepancy between the different results. In addition, the two waves of migration to the HCL also imply a consecutive generation of the subtypes.

The separate birth-dates and migration periods reveal the existence of temporal windows during which HC precursors can acquire different fates. For instance, the generation of stellate HCs could result from a combination of instructive signals acting at overlapping time points. Stellate HCs could be generated during an overlap of: a) a decreasing signal instructing early born HCs to express Ap2α and GABA, and b) an increasing signal instructing late-born HCs to express Isl1. The 6–10% abundance of Ap2α and GABA expression in the stellate HCs may suggest such a mechanism (Fig. 4T). In this scenario, later born Isl1+ HCs (i.e. the TrkA+ candelabrum HCs) would encounter only the second signal and develop along a third route. These potential signals remain to be characterized and may be either extrinsic, intrinsic or both. The signals seem not to be of a character that could be inhibited by follistatin since the subpopulations were not differentially affected by this treatment.

### Follistatin promotes the proliferation of HC-generating progenitor cells

In the developing retina extrinsic signals, exemplified by the TGFβ/BMP family members, are suggested to regulate nearly every aspect of retinal development, such as proliferation, migration, fate determination, cell death and functional maturation [26,27,29,58,59]. Follistatin, a potent inhibitor of activin and several other members of the TGFβ super family have previously been implicated to take part in the differentiation of retinal neurons [24,25,28]. For instance, a recent study indicated that follistatin could interfere with the generation of neurons in the developing retina, and also showed that follistatin was able to down-regulate the activin-dependent pSmad2 [28].

The central-to-peripheral temporal gradient of retinal development, where the central regions of the retina are more mature relative the peripherally located regions, is well characterized and makes the spatio-temporal aspect of retinal development an important factor to consider. Therefore, to understand how follistatin activity in the retina relates to our birth-dating of HCs as well as to the appearance of the early HC marker Lim1, we characterized the spatio-temporal follistatin expression. We found that follistatin expression followed a central-to-peripheral pattern and that with increasing age the immunoreactivity in the central retina decreased and an apparent wave-front of high follistatin expression moved towards to the periphery. The appearance of Lim1+ cells followed close behind this moving wave-front (Fig. 6C and 6D, black arrows). Combined with our qRT-PCR data demonstrating a dramatic 50-fold drop in follistatin mRNA levels that coincided with the time of HC birth, our observations show a correlation between low follistatin levels and HC generation.

In several cells and tissues, follistatin promotes proliferation by removing the inhibition of cell proliferation exerted by activin [42]. Our observations indicate that this is also true in the developing retina. For instance, activin injections in E3 embryos caused a robust decrease in BrdU incorporation (Fig. 7A–C), and during E2 (st14-18), which is a time marked by massive RPC proliferation in the developing retina, the relative levels of follistatin mRNA were extremely high (Fig. 6A). Combined, these observations indicate that follistatin stimulate proliferation in the retina, possibly by repressing activin signalling. Interestingly, the notion that follistatin stimulates proliferation may appear inconsistent with the observation that embryos injected with follistatin and analysed 22 h after the injection had fewer Lim1+ cells than control-treated embryos. Our interpretation is that the transient follistatin overexposure prevents HC-generating progenitor cells from producing post-mitotic cells, which turn on Lim1 expression. Instead, the affected progenitor cells may produce two mitotic daughters (not yet expressing Lim1) that will both inherit the capability to produce HCs. Thus, with a follistatin-induced increase of the HC-generating progenitor pool, the observed result will be a delayed onset of Lim1 expression, followed by an increase in the total number of HCs at a later time point (e.g. at E9), and this is what our results showed. Combined, these observations suggest that follistatin is important for early RPC proliferation in general and specifically for the proliferation and generation of HC progenitor/precursor cells (Fig. 10B). However, since the follistatin-phenotype at E9 (st35) most often was restricted to distinct regions of the retina, this indicates the existence of a spatio-temporal window during which HC-generating progenitor cells are susceptible to follistatin treatment which give rise to the described effects.

Moreover, embryos injected with follistatin at E3 and analysed at E9 (st35) had a significantly increased number of HCs. This increase was slightly more pronounced in the later-born Isl1+ HCs (approximately +50%) compared to the early Lim1+ HCs (approximately +40%) (Fig. 8, compare H and I, see also Additional file 3). Furthermore, the ectopic HCs scattered within the retina at st35 in follistatin-treated retinas were predominantly of the Isl1 subtype (Fig. 8B and 8E). One day later, at st36, these ectopic cells were no longer observed and we concluded that they had settled in the HCL (Fig. 8B and 8C), since the increased number of Prox1, Isl1 double positive HCs was similar in st35 and st36 retinas. Moreover, TUNEL staining was not detected in these cells (Fig. 8F). Our conclusion is that the ectopic and migrating HCs at st35 represent HCs that were produced in excess following follistatin treatment. This is in agreement with a model wherein follistatin cause HC-generating progenitor cells to proliferate beyond the normal period of HC generation, thus producing extra HCs that also migrate to the HCL.

Follistatin inhibits the action of ligands such as activin and other TGFβ-signalling molecules by binding them and thus preventing subsequent receptor activation. Hence, the inhibitory effects of follistatin depend on the presence of these ligands. Activin and its receptors are known to be present in the developing retina [24] and therefore activin stands out as a likely candidate through which follistatin exerts its inhibitory action. However, it cannot be excluded that the effects caused by follistatin treatment are generated by the inhibition of other TGFβ-signalling systems, or that the follistatin treatment may mediate its effects by indirectly influencing other cell signalling systems. Regardless of the pathway(s) by which follistatin affects retinal development at E3, our data suggest that the effects are exclusive to progenitor cells that generate HCs since we were unable to detect any differences in the number of other retinal cell populations at E9 following follistatin treatment at st23 (Fig. 9G–I, see also Additional file 3). This was perhaps surprising, considering the broad spectrum of ligands which follistatin can bind and inhibit, and also considering that classic lineage-tracing experiments have shown that early RPCs are homogeneously multipotent and capable of generating all retinal cell types [60,61].

The result that the other major retinal cell populations studied were not affected by follistatin might, however, be explained by one or more mechanisms. First, compared to HCs, most retinal cell populations are generated over a long period of time, which allows for secondary mechanisms to control proliferation and cell-fate decisions. This can be exemplified by post-mitotic amacrine cells and ganglion cells that through feedback inhibition to progenitor cells control the continued generation of these cell types, respectively [62,63]. In addition, apoptosis may also provide a mechanism by which, from E3 to E9, cell numbers are adjusted and corrected. Secondly, the brief overexposure-time of follistatin in our experimental setup may be insufficient to produce effects that would require long-term follistatin exposure, and may explain the inconsistency between our and Moreira and Adler's observations that following their continuous follistatin-overexposure, the region containing Ap2α amacrine cells decreased in thickness [28]. Thirdly, one may hypothesise that progenitor cells, which generate other retinal populations than HCs at E3 (for instance retinal ganglion cells and cone photoreceptors), rely on other signalling systems than follistatin to control their proliferation rate. Supporting this view are previous studies reporting that certain RPCs are biased to produce only a limited repertoire of neurons [56,64-66]. These three explanations are not mutually exclusive; however, based on our and other's data, we favour an explanation that assumes a degree of heterogeneity in the early RPC pool.

Horizontal cells are in several ways the odd ones out when compared to other retinal neurons. For example, HCs undergo bi-directional migration, and in contrast to most retinal cell populations, HC cell numbers are not adjusted by apoptosis, neither during early nor late development. As shown here, HCs are the only cell type that is affected by follistatin at E3, which suggests that progenitor cells that are competent to generate HCs are follistatin-sensitive, whereas progenitor cells that generate other early-born neurons, e.g. cone photoreceptors are not. In line with this, the existence of progenitor cells biased for HC generation have been indicated in mice [66] where Zhang et al. found using Cre-LoxP fate-mapping that an enhancer element of Pax6 was active in a restricted subset of RPCs, which almost exclusively generated HCs. The recent work by Godinho et al in zebrafish [56] and the lineage study in chick by Rompani and Cepko [57] both shows that there are progenitor cells fated to generate HCs by a final mitosis. Rompani and Cepko's data suggest that such dedicated HC progenitors are biased to generate two HCs of only one subtype.

Our results showed that the generation of both Lim1+ and Isl1+ subtypes were affected by the follistatin injections at st20-23 (Fig. 8G–I). Assuming that the effect by the early bolus injection of follistatin is transient and that it is washed out before the peak of Isl1+ HC generation, our data agree with an interpretation that progenitors are committed to a HC fate before their terminal mitosis or even before they undergo their last S-phase. This interpretation is consistent with the observation that pre-mitotic HC precursors in zebrafish bear morphological resemblance to HCs themselves and also express Ptf1a and Cx55.5 that are normally found in post-mitotic HCs [56].

## Conclusion

Axon-bearing and axon-less HC subtypes express Lim1 and Isl1, respectively. These two subtypes are born sequentially and their relative proportions are fixed early in development and are not adjusted by apoptosis. HCs are generated concomitantly with a decrease in endogenous follistatin levels. Adding exogenous follistatin at E3 clearly affected progenitor cells competent to generate HCs, and caused an over-production of HCs (Fig. 10B). Taken together, our data suggest that HC progenitors are sensitive to follistatin and become committed to produce Lim1+ and/or Isl1+ HC subtypes concomitantly with or early after their own generation.

## Methods

### Animals and in ovo cultures

Fertilized White Leghorn eggs (*Gallus gallus*) were obtained from Ova Produktion AB (Västerås, Sweden) and incubated at 38°C in a humidified incubator (Maino, Italy). Embryos were staged according to Hamburger and Hamilton [32]. Hatched chicks were kept at the animal facility at Evolutionsbiologiskt centrum, Uppsala. The animal work followed the EC guidelines and the ARVO statements for use of animals in ophthalmic and vision research. Experiments were scrutinized by the local animal ethics committee.

### Tissue preparation for immunohistochemistry

Whole eyes or a patch of the central-most retina were dissected and fixed in 4% PFA for 15–20 minutes, washed 10 minutes in PBS and cryoprotected in 30% sucrose for 3–4 hours before being frozen in OCT (Sakura). Dissected retina was flat-mounted onto nitrocellulose filters. Tissues were cryosectioned and collected on Superfrost Plus glasses (Menzel-Gläser). Flat-mounts were cut in 10 μm thick sections in a plane parallel to the filter producing sections through each retinal lamina. Cross-sections of the central retina were cut along the naso-temporal axis at 10 μm or 20 μm. Immunohistochemistry was preformed as described elsewhere [34].

### Antibodies

Primary antibodies; Prox1 (1:1000, rabbit, AB5475, Chemicon), Lim1/2 (1:20, mouse, 4F2, Developmental studies hybridoma bank (DSHB)), Ap2α (1:200, mouse, 3B5, DSHB), Pax6 (1:200, mouse, PAX6, DSHB and 1:4000, rabbit, AB5409, Chemicon), Isl1/2 (1:200, mouse, 40.2D6, DSHB, [67]) and Isl1 (1:10000, guinea pig, gift from Prof. J. Ericson), GABA (1:1000 rabbit, A2052, Sigma), Calretinin (1:1000, rabbit, 1741-1007, Anawa), TrkA (1:4000, rabbit, gift from Prof. L. Reichardt [68]), BrdU (1:500, mouse, B8434, Sigma), follistatin (1:100, goat, sc-23553, Santa Cruz) and Lim3 (1:200, mouse, 67.4E12, DSHB). Secondary antibodies were obtained from Vector Laboratories, Jackson Immunoresearch Laboratories or Molecular Probes.

### Microscopy and image processing

Samples were analysed using a Zeiss Axioplan2 microscope or a Zeiss LSM 510 confocal microscope, equipped with Axiovision software or LSM 510 imaging software (v3.2), respectively. Images were formatted, resized, enhanced and arranged for publication using Axiovision, LSM image browser and Adobe Photoshop. Graphs were made in Prism (v3.02, GraphPad software Inc.).

### In ovo embryo cultures

For the birth-dating experiment and follistatin/control injections, fertilized eggs were removed from the incubator on E2 or E3 and 3 ml egg white was aspirated from the egg to allow the yolk and embryo to detach from the inside of the eggshell. A small window was made in the eggshell above the embryo and re-sealed with transpore tape (3 M). Embryos were returned to the incubator and allowed to develop until the desired stage when manipulations were carried out. After manipulation, the windows were again re-sealed with tape and the eggs returned to the incubator where the embryos were allowed to develop until the time of analysis.

### Intra ocular follistatin injections

The eye of st20-23 (E3) embryos were microinjected with <0,2 μl follistatin (PeproTech, 1 μg/μl in PBS) using glass capillaries. Control injections contained vehicle. The solutions were supplemented with Fast Green (Kodak) in order to visualize the injected solution. Embryos were allowed to develop to st35-36 (E9–10) when injected eyes were processed for immunohistochemistry. The survival at st35 was typically 20–30% for both vehicle and follistatin injections.

Upon macroscopic inspection, injected eyes appeared normal when compared to un-injected and contralateral eyes. Deviations from normal appearance were rare. Histologically, the follistatin phenotype was not evenly spread over the retina but rather confined to distinct zones, so in order to select regions for cell counting (see below) the histological phenotype described in the Results part served as our criteria to determine if a retinal region had been affected by the follistatin. Un-affected regions were most often found in the periphery. Approximately 20% of the follistatin treated survivors did not produce a clear histological phenotype and were omitted from further analysis.

### Cell quantification and statistical analysis

The distribution of HC subtypes may vary with retinal location [43,69], thus all cell counts were performed in samples taken from the central retina. The region directly opposite to the lens and/or in proximity to the optic nerve exit was considered as central retina. HC counts were based on the assumption that Prox1 labels all HCs.

Cell quantification in developing embryos: 10 μm cross-sections of normal, follistatin or vehicle injected eyes were labelled using immunohistochemistry as described above. For HC quantification in st35 and st36 embryos, fluorescence micrographs of sections with Prox1 labelling in combination with Lim1 or Isl1 were acquired using the 40× objective on an Axioplan2 microscope. The number of cells positive for Prox1 alone, or Prox1, Lim1 double positive-, and Prox1, Isl1 double positive cells was determined from two series of injections using a minimum of 4 individuals surviving the injections per stage as indicated in figures and legends. A minimum of 4 micrographs from 4 retinal sections per animal were used for cell quantification, which was facilitated by Photoshop and ImageJ (NIH). Statistical analyses were performed in Prism (v3.02, GraphPad software Inc.) using the Mann-Whitney test and p-values < 0.05 were considered significant (see also Additional file 3). For quantification of other cell populations than HCs following follistatin treatment, the same retinas used for HC quantification above were immuno-labelled using various markers. Cell counting and statistical analysis was done as described above.

To test the effects of follistatin injections on the number of Lim1+ cells early in development, st21, st22 and st23 eyes were injected with follistatin or vehicle as described above and processed for Lim1 immunohistochemistry 22 h after the injections (number of animals analysed per stage is indicated in Fig. 6E and Additional file 3). Images from central retina were obtained from 5 sections per embryo using a 20× objective and the number of Lim1+ cells was quantified using Photoshop and ImageJ. The area of the cross-sections was determined using Axiovision and the density of Lim1+ cells in each section was expressed in cells/mm^2 ^retina. Statistical analyses were performed in Prism (v3.02, GraphPad software Inc.) using the Mann-Whitney test and p-values < 0.05 where considered significant (see also Additional file 3).

HC subtype quantification at P0: Z-stacks (~0, 4 μm intervals) of the HCL were obtained from 20 μm thick retinal cross sections stained for Lim1, Isl1, Ap2α combined with Prox1, GABA, calretinin or TrkA using a 63× objective in a LSM confocal microscope. Each Z-stack contained on average 75 HCs. From 3 P0 chick retinas, at least 4 regions in direct proximity to the optic nerve exit were photographed for each staining combination and the fractions of single- or double positive cells within the stack were counted manually using the LSM 510 imaging software.

### Birth-dating of horizontal cell subtypes

Carefully staged embryos ranging from st19 to st33 received a single dose of tritiated deoxy-thymidine ([^3^H]-dT, 1 μCi/μl, TRK758, Amersham). Stage 19–26 embryos received 15 μCi, and st27-33 embryos received 25 μCi. A minimum of three embryos per stage was used (except stages 32 and 33, see Fig. 2). Embryos were allowed to develop until st35, when the central most piece of retina was punched out using a sharpened metal tube 4.5 mm in diameter. Each retina was fixed (10 min, 4% PFA), washed (10 min, PBS) and dissociated to a single-cell suspension in 0,25% trypsin. Cells were pelleted and resuspended in 1 ml PBS (approximately 10^6 ^cells/ml). The cell suspensions were plated onto 8-well slides (PH-098, NovaKemi AB, Sweden, 50 μl/well) and air-dried. Slides were processed for Prox1 or Lim1 immunocytochemistry, using the Vectastain Elite ABC-kit (PK-6102) and the 3,3'-diaminobensidine (DAB) peroxidase substrate kit (SK-4100) from Vector Laboratories, according to the manufacturers instructions. Slides were pre-treated in 0,5% Cobalt(II)Chloride before the DAB reaction to maintain DAB staining through the autoradiographic processing [70]. Slides were dipped in EM Hypercoat Emulsion (RPN41, GE Healthcare) and left to expose in darkness for 6–8 days at 4°C before being developed using Kodak D-19 and Kodak Fixer (P6557, Sigma). Slides were mounted in Entellan (Merck) and analysed by light microscopy. The optimal exposure time was determined for each stage and the threshold that was considered as a positive [^3^H]-dT labelled cell was determined for each individual experiment by identifying the most heavily [^3^H]-dT labelled cells per sample and using those cells as reference (see Additional file 1). On average, 260 immunolabelled cells per stage and antibody were counted.

In addition to the round HCs with strong nuclear Prox1 labelling, weak Prox1 labelling is found within the INL at st35 in elongated cells that are not HCs [33]. Therefore, a distinction between Prox1+ HCs and non-HCs was made; elongated faintly DAB-labelled cells were considered non-HCs whereas rounded intensely nuclear DAB-labelled cells were considered to be HCs (Additional file 1, compare cells in A-B with cells in C). Only cells with intense nuclear Prox1 labelling were counted.

Since Lim1+ HCs constitute half of all Prox1+ HCs at st35 (Fig. 1B), the raw data obtained from Lim1 cell counts were divided by two. For example, if from one stage 15 out of 25 Lim1+ cells were also [^3^H]-dT+, then this indicated that 60% of the Lim1 expressing HCs were born around this time. However, since Lim1+ HCs at the time of analysis (st35) constitute half of all HCs, this number actually corresponds to 30% of all HCs with respect to Prox1. Thus, to relate the fraction of [^3^H]-dT+ Lim1 cells to the fraction of [^3^H]-dT+ Prox1 cells at the same stage, 30% (and not 60%) would be the Lim1 percentage plotted in figure 2 for that specific stage (see also Additional file 3). Moreover, since we were unable to determine the birth-curve of Isl1+ HCs directly, the numerical difference between [^3^H]-dT+ Prox1 and Lim1 fractions were plotted as a line to represent the birth-curve of Isl1+ HCs. Statistical analysis using the Student's t-tests were preformed at every stage to test whether the [^3^H]-dT+ Prox1 and Lim1 fractions were significantly different (* p < 0.05).

### TUNEL in combination with immunohistochemistry

To analyse cell death during retinal development we modified the protocol supplied with the DeadEnd Fluorometric TdT-mediated-dUTP-nick-end-labelling (TUNEL) system (G3250, Promega) to be combined with immunohistochemistry. Sections were labelled for Prox1 and fixed in 4% PFA prior to TUNEL staining, and secondary antibodies were added after the TUNEL reaction. Retinas from all stages between st20 and st36 but also stages 39, 41, 42, 44, P0, P4 and P7 were analysed. In addition, follistatin and vehicle-treated retinas were analysed with respect to Prox1 and TUNEL at st35-36 (E9–10).

### Quantitative real time PCR

Total RNA was extracted from embryonic tissues using the Trizol reagent (Invitrogen). Retinal tissue form stages 14, 16, 18, 21, 23, 25, 28, 30, 35, 38, 42 and 45 were used. For st14-18, optic cups from 20–40 embryos were collected and pooled with some contamination from lens and retinal pigment epithelium. For embryos older than st18, 2–14 retinas were collected and pooled. Two batches of cDNA were prepared separately from 1 μg of RNA using GeneAmp^® ^(Applied Biosystems). Samples were run in triplicate on both cDNA batches using IQ™ SyBr^® ^Green Supermix (Biorad) and normalized to β-actin and TATA-box binding protein. Control reactions containing primers but no RNA were analysed in parallel. Primer sequences were; β-actin (NM205518; 5'-aggtcatcaccattggcaatg-3' and 5'-cccaagaaagatggctggaa-3'), TATA-box binding protein (D83127; 5'-tagcccgatgatgccgtat-3' and 5'-gttccctgtgtcgcttgc-3'), follistatin (NM205200, 5'-cgtcaacgacaacacgctctt-3' and 5'-ccaggtccacagtccacattct-3'), follistatin-like 1 (NM204638, 5'-attgtgagctgcaccgagatg-3' and 5'-aactggactcgcagctggatt-3').

### BrdU-assay in early activin-treated retinas

To test the effect of activin on retinal precursor cell proliferation we injected st23 embryos with activin (PeproTech, 25–50 μg/ml in 5 mM citric acid) or vehicle as described above. Five hours after injection, 100 μl of 10 mg/ml BrdU (Sigma) was injected to the egg and 22 hours after the initial injection, embryos were analysed for BrdU incorporation by immunohistochemistry, with the modification that eyes were fixed in PFA for 1 hour. The number of BrdU+ cells following activin (n = 5) or vehicle treatment (n = 4) were quantified from a minimum of 4 cross-sections per embryo using Photoshop and ImageJ. The area of the cross-sections of retina that were sampled for labelled cells was determined using Axiovision and the density of BrdU+ cells in each cross-section was expressed in cells/mm^2 ^retina. Statistical analyses was performed in Prism (v3.02, GraphPad software Inc.) using the Mann-Whitney test, p-value < 0.05 was considered significant.

## Authors' contributions

PHE designed the experimental work, carried out immunostainings, [^3^H]-dT birth-dating analysis, apoptosis analysis, BrdU-assay, did most intraocular injections and most cell counting, analysed the data, wrote the manuscript and made the figures. HB performed the qRT-PCR analysis and contributed to the manuscript. ML carried out intraocular injections and contributed to the manuscript. SML carried out [^3^H]-dT birth-dating analysis and contributed to the manuscript. FH conceived of the study, designed and coordinated the work and wrote the manuscript. All authors read and approved its final version.

## Supplementary Material

Additional file 1 Representative cells from birth-dating experiment using [^3^H]-dT in combination with Prox1. High power bright-field micrograph of dissociated st35 retinal cells labelled for Prox1 (DAB colorimetric staining, brown) and processed for autoradiographical detection of [^3^H]-dT incorporation. Click here for file

Additional file 3 Data presented in figures 2 and 6 to 9. Data used for making graphs in figures 2, 6, 7, 8 and 9. Click here for file

Additional file 2 Follistatin expression in st34 retina. Follistatin immunoreactivity (red) in a st34 retina is restricted to the ganglion cell layer (gcl) and to certain cells located on the inner-most rim of the inner nuclear layer (inl). This pattern together with the observation that follistatin treatment cause a thinning of the inner plexiform layer (this study and ref [28]), and our data demonstrating that follistatin mRNA levels increase from st35 and beyond suggest that follistatin also has a function in the development, organization and/or establishment of the inner plexiform layer. Scale bar: 20 μm. Click here for file
