# Supplementary figures and images for: Axon-bearing and axon-less horizontal cell subtypes are generated consecutively during chick retinal development from progenitors that are sensitive to follistatin
Source: BMC Dev Biol. 2008 Apr 25;8:46. doi: 10.1186/1471-213X-8-46 (PMC2386784; doi:10.1186/1471-213X-8-46)

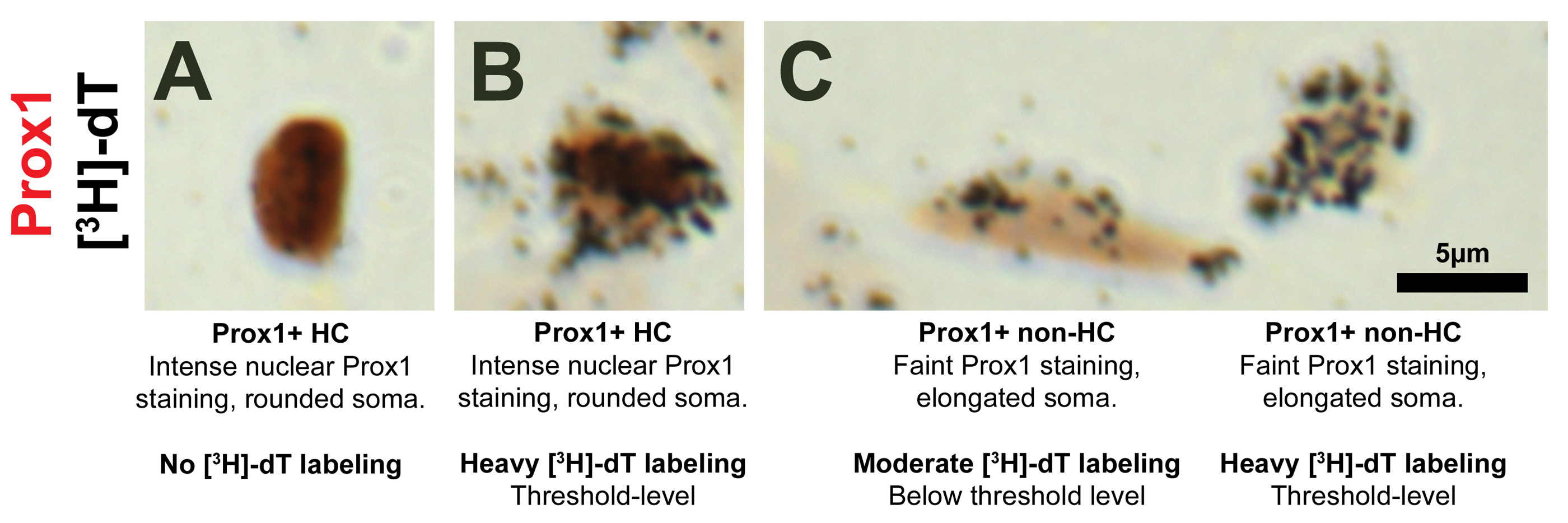

Supplement: Additional file 1 — Representative cells from birth-dating experiment using [3H]-dT in combination with Prox1. High power bright-field micrograph of dissociated st35 retinal cells labelled for Prox1 (DAB colorimetric staining, brown) and processed for autoradiographical detection of [3H]-dT incorporation. [file 1471-213X-8-46-S1.tiff]

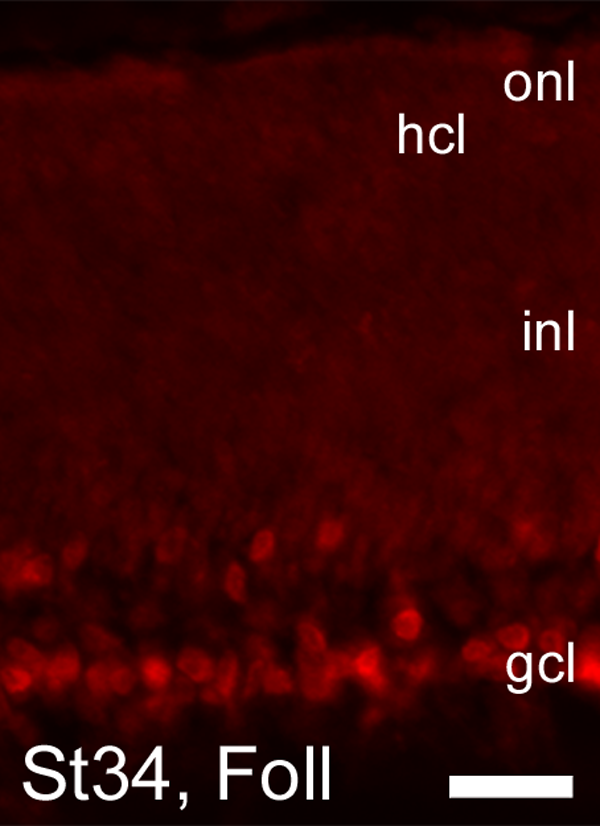

Supplement: Additional file 2 — Follistatin expression in st34 retina. Follistatin immunoreactivity (red) in a st34 retina is restricted to the ganglion cell layer (gcl) and to certain cells located on the inner-most rim of the inner nuclear layer (inl). This pattern together with the observation that follistatin treatment cause a thinning of the inner plexiform layer (this study and ref [28]), and our data demonstrating that follistatin mRNA levels increase from st35 and beyond suggest that follistatin also has a function in the development, organization and/or establishment of the inner plexiform layer. Scale bar: 20 μm. [file 1471-213X-8-46-S2.tiff]
