# Supplementary material for: Axon-bearing and axon-less horizontal cell subtypes are generated consecutively during chick retinal development from progenitors that are sensitive to follistatin
Source: BMC Dev Biol. 2008 Apr 25;8:46. doi: 10.1186/1471-213X-8-46 (PMC2386784; doi:10.1186/1471-213X-8-46)
Supplement: Additional file 3 — Data presented in figures 2 and 6 to 9. Data used for making graphs in figures 2, 6, 7, 8 and 9. [file 1471-213X-8-46-S3.pdf]

Data presented in figures 2 and 6 to 9

Fig 2: Birthdating of horizontal cells

Expressed as % immunolabelled cells also positive for 3H-dT (analysed at st35)

Injection stage	Prox1	± s.d.	Lim1	± s.d.	Difference (=Isl1+ HC)	t-test p<0.05
19	34.8	8.1	29.9	3.9	4.9	n
20	48.4	6.7	40.5	4.3	7.9	n
21	58.8	11.5	44.2	3.3	14.6	y
22	57.2	9.5	33.8	4.7	23.4	y
23	52.9	8.5	21.0	4.3	31.9	y
24	50.9	5.5	15.4	3.8	35.5	y
25	41.7	6.6	9.3	2.8	32.4	y
26	43.2	3.8	8.6	3.0	34.6	y
27	27.9	6.4	5.0	3.8	22.9	y
28	26.6	3.3	6.3	2.8	20.3	y
29	14.9	3.9	6.2	2.0	8.7	y
30	11.2	2.2	4.0	2.1	7.2	y
31	7.0	2.3	2.5	2.6	4.5	y
32	2.6	3.0	0.8	1.0	1.8	n
33	1.6	2.0	0.4	0.8	1.2	n

Fig 6A: qRT-PCR

Stage	Relative values Follistatin	FSTL1
st14	2689.24	3.10
st16	593.64	3.36
st18	246.28	3.85
st21	63.87	4.78
st23	22.77	4.30
st25	5.68	4.99
st28	1.00	4.54
st30	1.43	3.36
st35	7.45	1.82
st38	157.44	1.14
st42	145.83	1.00
st45	110.76	1.30

Fig. 6E: Lim1+ cells 22h after follistatin injection

Expressed as Lim1+cells/mm2

Stage	st21		st22		st23	
Treatment	**Control**	**Follistatin**	**Control**	**Follistatin**	**Control**	**Follistatin**
n animals	4	5	6	6	4	7
Median	245.0	192.4	274.0	192.4	305.6	236.3
p-value (MW)	0.0159		0.0022		0.0424	
**Mean values ± s.d.**						
Mean value	248.7	185.2	271.8	199.0	293.2	234.0
± pooled s.d.	36.9	24.2	33.4	27.1	40.6	21.9
**Expressed as % of average mean value**						
Mean value	100.0	74.5	100.0	73.2	100.0	79.8
± s.d.	6.9	6.0	4.4	11.8	14.1	8.8

Fig. 7C: BrdU+ cells 22h after activin injection at st23

Expressed as BrdU+cells/mm2

Treatment	Control	Activin
n animals	4	5
Median	1443.9	366.2
p-value (MW)	0.0159	
**Mean values ± s.d.**		
Mean value	1468.8	420.9
± pooled s.d.	153.9	78.7

Fig. 8G-I: Number of horizontal cells at st35 after follistatin injection at E3

Expressed as cells/ 40x visual field

8G: Prox1

Treatment	Control		Follistatin			
Stage	**st21**	**st23**	**st20**	**st21**	**st22**	**st23**
n animals	5	4	5	4	4	6
Median	53.5	58.9	71.1	70.6	71.4	75.5
Mann-Whitney p-value (vs. ctrl st21)			0.0079	0.0159	0.0159	0.0043
Mann-Whitney p-value (vs. ctrl st23)			0.0159	0.0286	0.0286	0.0095
**Mean values ± s.d.**						
Mean value	53.0	59.2	73.3	72.3	71.9	79.3
± pooled s.d.	4.6	6.3	7.7	6.9	8.0	9.9
**Expressed as % of average mean value**						
Mean value	100.0		131.5	129.5	128.9	142.2
± s.d.	6.6		12.2	14.3	4.9	16.0

8H: Lim1

Treatment	Control		Follistatin			
Stage	**st21**	**st23**	**st20**	**st21**	**st22**	**st23**
n animals	5	4	5	4	4	6
Median	26.0	25.6	36.0	36.9	36.8	39.4
Mann-Whitney p-value (vs. ctrl st21)			0.0079	0.0159	0.0159	0.0043
Mann-Whitney p-value (vs. ctrl st23)			0.0159	0.0286	0.0286	0.0190
**Mean values ± s.d.**						
Mean value	26.1	26.7	35.0	36.6	36.4	38.3
± pooled s.d.	2.6	2.4	4.0	3.5	4.4	5.0
**Expressed as % of average mean value**						
Mean value	100.0		133.0	139.1	138.4	145.4
± s.d.	6.2		8.8	19.5	5.2	22.0

8I: Isl1

Treatment	Control		Follistatin			
Stage	**st21**	**st23**	**st20**	**st21**	**st22**	**st23**
n animals	5	4	5	4	4	6
Median	25.8	29.6	40.3	37.3	43.8	44.4
Mann-Whitney p-value (vs. ctrl st21)			0.0079	0.0159	0.0159	0.0043
Mann-Whitney p-value (vs. ctrl st23)			0.0159	0.0286	0.0286	0.0095
**Mean values ± s.d.**						
Mean value	26.4	29.3	43.2	39.1	41.1	44.8
± pooled s.d.	2.6	4.0	6.2	4.7	5.6	7.6
**Expressed as % of average mean value**						
Mean value	100.0		156.0	141.2	148.4	161.8
± s.d.	7.2		33.9	19.3	11.5	28.8

Fig. 9G-I: Number of various cell types at st35 after follistatin injection at st23

Expressed as cells/ 40x visual field

**9G: Photoreceptors**				
Cell type	**Isl1 PRs**		**Lim3 PRs**	
Treatment	**Control**	**Follistatin**	**Control**	**Follistatin**
n animals	4	6	3	4
Median	96.6	99.0	115.3	108.0
p-value (MW)	0.914		0.400	
**Mean values ± s.d.**				
Mean value	97.9	104.2	111.4	105.2
± pooled s.d.	7.6	14.2	7.4	9.8

**9H: Bipolar cells**				
Cell type	**Isl1 BPs**		**Lim3 BPs**	
Treatment	**Control**	**Follistatin**	**Control**	**Follistatin**
n animals	4	6	3	4
Median	121.4	128.5	228.8	231.5
p-value (MW)	0.610		0.857	
**Mean values ± s.d.**				
Mean value	121.5	115.0	234.4	234.1
± pooled s.d.	11.8	15.9	14.9	21.8

**9I: Ganglion cells and amacrine cells**				
Cell type	**Isl1 RGCs/ACs**		**Ap2α ACs**	
Treatment	**Control**	**Follistatin**	**Control**	**Follistatin**
n animals	4	6	3	4
Median	109.6	119.4	242.0	244.5
p-value (MW)	0.257		0.629	
**Mean values ± s.d.**				
Mean value	107.6	117.6	238.9	245.5
± pooled s.d.	10.2	19.2	14.3	17.5

Note on "pooled s.d.-values":

The ""± pooled s.d."- values were calculated by pooling the variances of all individuals adhering to a specific stage and treatment, using the standard formula:

$$PooledSD = \sqrt{\frac{\left((n_1-1)s_1^2 + (n_2-1)s_2^2 \ldots\right)}{\left((n_1-1) + (n_2-1)\ldots\right)}}$$

n= number of observations for each individual (eg. Individual 1= 4 observations, individual 2= 8 observations)
s= s.d. for those 4 or 8 observations, respectively

Thus, each s.d. value that belongs to an individual is weighed based on the number of observations that gave the value. (e.g. A s.d.-value based on 4 observations is given less weight than a s.d.-value based on 8 observations).

P-values were calculated in Prism using either the Student's t-test (Fig. 2), or the Mann-Whitney (Figs. 6-9, indicated by MW).
